# A Novel Mitochondrial Serine *O*-Acetyltransferase, OpSAT1, Plays a Critical Role in Sulfur Metabolism in the Thermotolerant Methylotrophic Yeast *Ogataea parapolymorpha*

**DOI:** 10.1038/s41598-018-20630-8

**Published:** 2018-02-05

**Authors:** Ji Yoon Yeon, Su Jin Yoo, Hiroshi Takagi, Hyun Ah Kang

**Affiliations:** 1 Department of Life Science, Chung-Ang University, Seoul, 06974 Korea; 2 Graduate School of Biological Sciences, Nara Institute of Science and Technology, Nara, 630-0192 Japan

## Abstract

In most bacteria and plants, direct biosynthesis of cysteine from sulfide via *O*-acetylserine (OAS) is essential to produce sulfur amino acids from inorganic sulfur. Here, we report the functional analysis of a novel mitochondrial serine *O*-acetyltransferase (SAT), responsible for converting serine into OAS, in the thermotolerant methylotrophic yeast *Ogataea parapolymorpha*. Domain analysis of *O. parapolymorpha* SAT (OpSat1p) and other fungal SATs revealed that these proteins possess a mitochondrial targeting sequence (MTS) at the N-terminus and an α/β hydrolase 1 domain at the C-terminal region, which is quite different from the classical SATs of bacteria and plants. Noticeably, OpSat1p is functionally interchangeable with *Escherichia coli* SAT, CysE, despite that it displays much less enzymatic activity, with marginal feedback inhibition by cysteine, compared to CysE. The *Opsat1*Δ-null mutant showed remarkably reduced intracellular levels of cysteine and glutathione, implying OAS generation defect. The MTS of OpSat1p directs the mitochondrial targeting of a reporter protein, thus, supporting the localization of OpSat1p in the mitochondria. Intriguingly, the OpSat1p variant lacking MTS restores the OAS auxotrophy, but not the cysteine auxotrophy of the *Opsat1*Δ mutant strain. This is the first study on a mitochondrial SAT with critical function in sulfur assimilatory metabolism in fungal species.

## Introduction

Cellular requirements for sulfur, an essential element for all living organisms, can be fulfilled by the uptake of sulfur-containing amino acids or by the assimilation of inorganic sulfur into organic compounds such as cysteine and homocysteine, which are used for further biosynthesis of the tripeptide glutathione (GSH) and methionine, respectively^1^. Cysteine can be synthesized via the sulfur assimilation pathway in microorganisms and plants, except animals, which do not have assimilatory mechanisms for inorganic sulfur^2^. In bacteria such as *Escherichia coli* and *Salmonella typhimurium*^3–5^, *de novo* synthesis of cysteine from sulfide occurs by two sequential reactions mediated by serine *O*-acetyltransferase (SAT; EC 2.3.1.30) and *O-*acetylserine sulfhydrylase (OASS; EC 2.5.1.47). SAT catalyzes the synthesis of *O*-acetylserine (OAS) from acetyl-CoA (AcCoA) and L-serine (L-Ser). Synthesized OAS is sequentially condensed with sulfide by OASS to form cysteine. This two-step reaction is called the OAS pathway. SAT is the key enzyme in the cysteine biosynthesis pathway because its feedback inhibition by cysteine regulates the biosynthesis of cysteine in bacteria^5^. The OAS pathway also plays key roles in cysteine biosynthesis in the sulfur assimilatory metabolism of plants^1,6^. Notably, some plant species, such as *Arabidopsis thaliana* have three SAT isoforms—SAT-c (cytosol), SAT-p (plastid), and SAT-m (mitochondria), which differ in their subcellular localization and amino acid sequences^7^. Each isoform possesses different enzymatic activity depending on its subcellular localization^8^.

**Figure 1:**
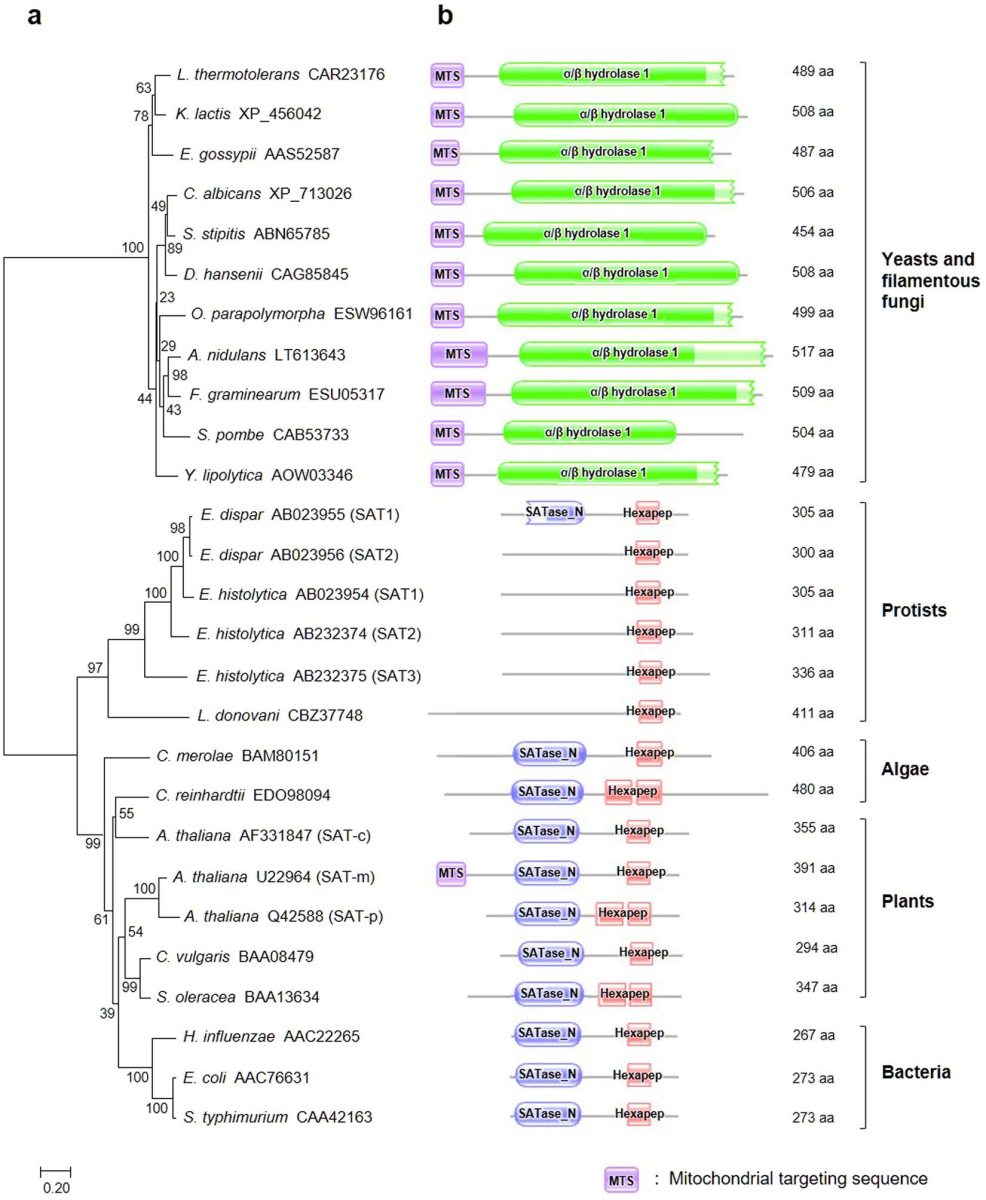
Phylogenetic and domain analysis of SATs from various organisms. (**a**) Phylogenetic tree of twenty-seven SAT proteins from representative organisms. Amino acid sequences of SAT were obtained from the GenBank database and subjected to phylogenetic tree construction using the Neighbor joining (NJ) method built in the MEGA7 software. GenBank accession numbers are indicated and some isoforms are written in parenthesis. The scale bar represents 0.2 amino acid substitutions per site. Bootstrap values (100 replicates) are shown next to the branches. (**b**) Predicted domain structure of SAT proteins. Mitochondrial targeting sequence (MTS) was predicted using the mitochondrial targeting prediction tool MITOPROT II^53^, and protein domain analysis was carried out using the Pfam database. SATase_N indicates N-terminal domain of SAT and Hexapep stands for hexapeptide repeat domain.

Plant, bacterial, and algal SATs show high similarity in domain structure, with all of them carrying the N-terminal domain of serine acetyltransferase (SATase_N), which is associated with enzymatic activity, and the C-terminal hexapeptide repeat domain, which is involved in the formation of the SAT-cysteine synthase (CS; EC 2.5.1.47) complex (please note that CS and OASS catalyze the same reaction in which OAS and S^2−^ are converted into cysteine and acetate). Some parasitic protists, such as the *Entamoeba* genus, *Trypanosoma* genus, and *Leishmania donovani*, have SAT for cysteine biosynthesis from sulfide^9–11^. Interestingly, the enteric parasite *Entamoeba histolytica* possesses three isoforms of SAT (i.e., SAT1, SAT2, and SAT3) but, contrary to those of *A. thaliana*, all of them co-localize in the cytoplasm. Protozoan SATs contain only hexapeptide repeat domain^10,12^, except for *E. dispar* SAT1, which harbors the SATase_N domain as we predicted (Fig. 1).

The OAS pathway is also reported in certain fungal species^13,14^. However, in yeast and fungal species, cysteine biosynthesis can be also achieved independently from the OAS pathway. Sulfide is condensed with *O*-acetylhomoserine to generate homocysteine, which is converted to cystathionine and then to cysteine via reverse trans-sulfuration pathway. The filamentous fungi *Aspergillus nidulans* and *Neurospora crassa* employ both pathways for cysteine biosynthesis^14,15^, whereas the budding yeast *Saccharomyces cerevisiae* does not possess the OAS pathway^16,17^. Previous studies concluded that detectable SAT and OASS do not constitute an cysteine biosynthetic pathway and that cysteine is exclusively synthesized via cystathionine by cystathionine β-synthase and cystathionine γ-lyase^18,19^. Thus, differently from plants and bacteria in which cysteine is the central precursor of all organic sulfur molecules, homocysteine is utilized as the main precursor for both cysteine and methionine synthesis in most fungal species. While various SATs in bacteria, parasites, and plants have been intensively investigated for their function and regulation, fungal SATs have been unexplored yet. To date, only two SATs from the filamentous fungi *A. nidulans* and *Fusarium graminearum* have been reported. The deletion mutants *cysA* of *A. nidulans* and *FgSAT* of *F. graminearum*, which encode SATs, grow normally on minimal medium because of the activation of alternative pathways involving homocysteine^20,21^.

The thermotolerant methylotrophic yeast *Hansenula polymorpha* is characterized by high tolerance to various stresses induced by heavy metals, xenobiotics (drugs), and environmental pollutants^22,23^. *H. polymorpha* has attracted much attention as an industrial yeast strain for various biotechnological applications^24^, and has been used as a host system for the production of various recombinant proteins ranging from industrial enzymes to therapeutics^25^. Since methylotrophic yeasts are obligatorily dependent on GSH for oxidation and detoxification of formaldehyde, a toxic methanol oxidation intermediate, *H. polymorpha* is a promising host strain for high-level production of GSH^26,27^. Three representative *H. polymorpha* strains of independent origin, DL-1 (ATCC26012), CBS4732 (ATCC34438), and NCYC495 (ATCC14754), are widely used for basic and applied researches. Recently, based on ribosomal DNA sequences and several taxonomic criteria, the *H. polymorpha* DL-1 strain has been reclassified as *O. parapolymorpha*, while the other two *H. polymorpha* strains, CBS4732 and NCYC495, have been designated as *O. polymorpha*^28,29^. We previously reported on the unique feature of the sulfur pathway of *O. parapolymorpha*, which exploits the OAS pathway as the only pathway for the synthesis of sulfur amino acids from inorganic sulfur. The *O. parapolymorpha* gene *OpSAT1* is homologous to *A. nidulans cysA* and its deletion was shown to generate the cysteine auxotrophic phenotype of the *OpSAT1*Δ-null mutant (*Opsat1*Δ) strain^13^.

In this study, we systematically analyzed the structural characteristics and physiological function of the *O. parapolymorpha* Sat1p (OpSat1p). We showed that OpSat1p is functionally interchangeable with *E. coli* CysE, despite their different structural organization and low sequence identity. Moreover, its localization at the mitochondria is required for full activity. Our data present OpSat1p as a novel mitochondrial SAT with a pivotal role in sulfur assimilatory metabolism in *O. parapolymorpha*.

## Results

### Distinctive features of OpSat1p as a novel SAT

To obtain comprehensive information on the structural features and evolutionary origin of OpSat1p, we constructed the phylogenetic tree of SATs from various organisms, including yeast, filamentous fungi, protists, algae, plants, and bacteria. Of the 27 SATs identified, three isoforms belong to *E. histolytica* (SAT1, SAT2, SAT3), three to *A. thaliana* (SAT-m, SAT-p, SAT-c), and two to *E. dispar* (SAT1, SAT2). The phylogenetic tree clearly revealed the separation of the SATs of yeasts and filamentous fungi from those of the other groups (Fig. 1a). Moreover, the SATs of yeast and filamentous fungi showed unique domain structure, quite different from that of classical SATs (Fig. 1b). Notably, all the SATs of yeasts and filamentous fungi were predicted to possess a mitochondrial targeting sequence (MTS) at their N-terminus and the α/β hydrolase 1 domain at the C-terminal region. In contrast, SATs from algae, bacteria and plants contain the N-terminal domain of SAT (SATase_N) and a hexapeptide repeat domain. Most protist SATs only contain the hexapeptide repeat domain, with the exception of *E. dispar* SAT1. This clearly indicates that the SATs of yeasts and filamentous fungi have evolved divergently from those previously characterized from other organisms.

The amino acid sequences of various SATs from fungi showed high identity (49.6–65.6%), revealing the presence of a highly conserved α/β hydrolase 1 domain (Fig. S1). However, the sequence identity among fungal SATs and those from bacteria and plants was quite low (9.4–10.2%). Multiple amino acid sequence alignment of representative fungal SATs (i.e., *O. parapolymorpha* Sat1p and *A. nidulans* CYSA) with those of bacteria and plants (i.e., *A. thaliana* SAT-m, *E. coli* CysE, *E. histolytica* SAT1, and *C. reinhardtii* SAT) showed a few conserved amino acid sequences, particularly located in the hexapeptide repeat domain (Fig. S2). In the hexapeptide sequence with a consensus sequence of {V,L,I}-G-X-X-X-X, the amino acid residue at the first position of each hexad is Val, Leu, or Ile and the second is often Gly^30^. Considering that the α/β hydrolase domain is associated with catalytic activity^31^ and that the hexapeptide repeat domain is a bifunctional domain, associated with both SAT/OAS-TL interaction and catalytic activity in *A. thaliana*^16^, it might be speculated that the conserved amino acids are involved in the SAT catalytic activity for conversion of serine into OAS. The physiological significance of these conserved amino acids between fungal and classical SATs needs further study.

### *In vitro* SAT activity of *O. parapolymorpha* Sat1p

**Figure 2:**
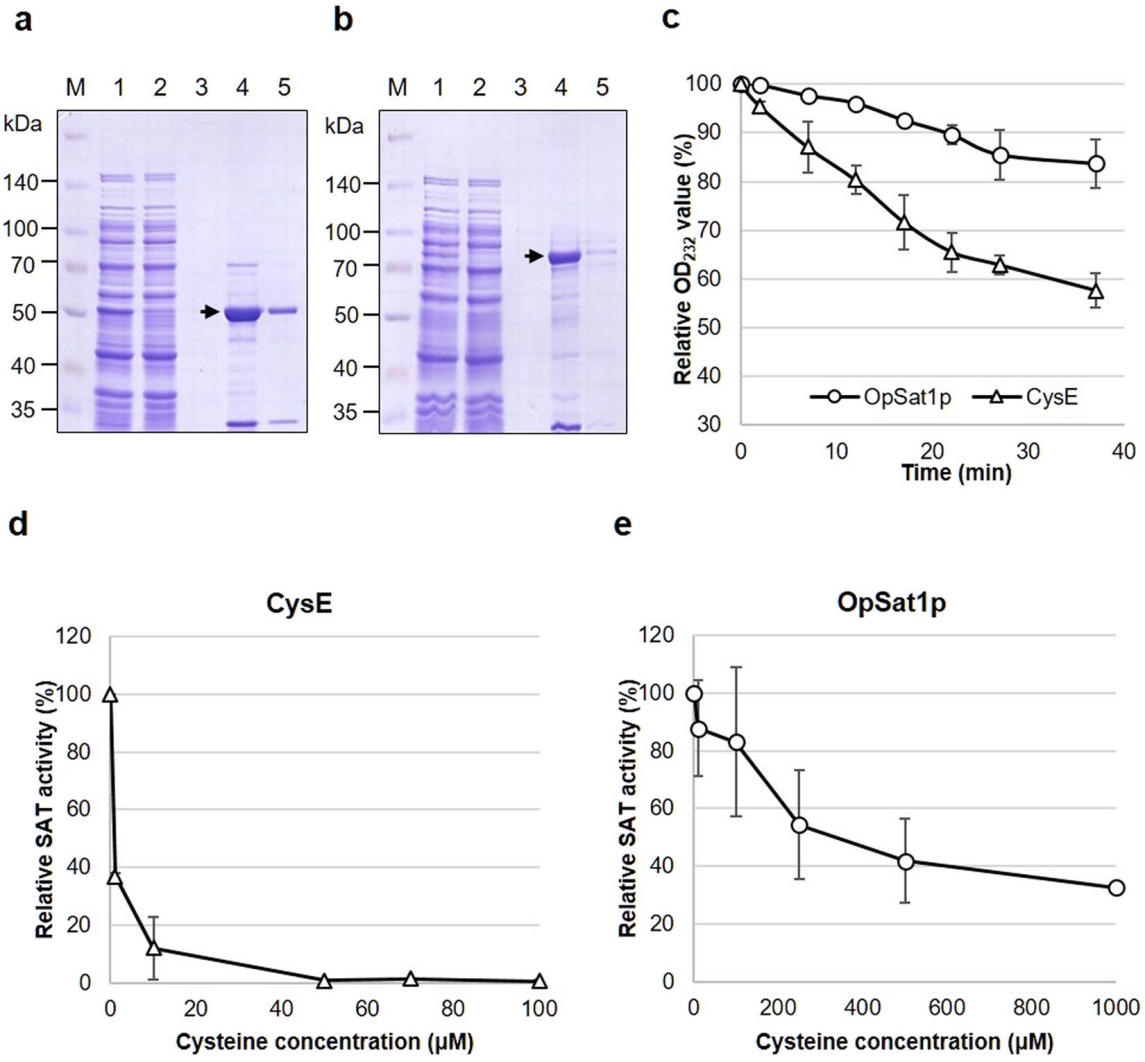
Purification and *in vitro* activity analysis of the recombinant OpSat1 and CysE proteins expressed in *Escherichia coli*. (**a**) Purification of GST-CysE. Lane 1, cell lysates from *E. coli* cells harboring the *cysE* expression vector pGEX4T1-cysE. Lane 2, Flow-through. Lane 3, wash with binding buffer. Lanes 4 and 5, eluted GST-CysE protein. The protein band of GST-CysE (55.8 kDa) is indicated by an arrow on the 8% SDS-polyacrylamide gel. (**b**) Purification of GST-OpSat1p. Lane 1, cell lysates from *E. coli* cells harboring the *OpSAT1* expression vector pGEX4T1-OpSAT1. Lane 2, Flow-through. Lane 3, wash with binding buffer. Lanes 4 and 5, eluted GST-OpSat1 protein. The protein band of GST-OpSat1p (80.6 kDa) is indicated by an arrow on the 8% SDS-polyacrylamide gel. (**c**) SAT activity of the purified *O. parapolymorpha* GST-Sat1p and *E. coli* GST-CysE . The reaction mixture (400 μL) contained 100 mM potassium phosphate buffer (pH 7.5), 0.1 mM acetyl-CoA, 1 mM L-serine, and 0.15 μg of GST-CysE or 15 μg of GST-OpSat1 protein. The decrease of OD_232_ absorbance caused by the hydrolysis of the thioester bond in acetyl-CoA was measured to determine SAT activity. (**d**,**e**) Feedback inhibition of SAT activity by L-cysteine. △, CysE; ○, OpSat1p. Cysteine was added to the enzyme mixture at the indicated concentrations and enzyme activity was assayed in triplicate.

To investigate the *in vitro* activity of OpSat1p in comparison with *E. coli* CysE, both SATs were expressed as recombinant proteins fused with glutathione *S*-transferase (GST) at the N-terminus. Additionally, the GST-fused SATs were tagged with a C-terminal 6x-his to facilitate the detection and purification of the recombinant constructs. The GST-CysE and GST-OpSat1p fusion proteins were expressed as soluble proteins with the size of 55.8 kDa and 80.6 kDa, respectively, and purified using a Ni-NTA column. The purity of the GST fusion proteins was determined on a sodium dodecyl sulfate polyacrylamide gel electrophoresis (SDS-PAGE) gel stained with Coomassie Brilliant Blue (Fig. 2a and b). Both GST-CysE and GST-OpSat1p were subsequently used for the *in vitro* assay of SAT activity based on the decrease rate of OD_232_ value, which reflects the consumption rate of the acetyl-CoA substrate. As shown in Fig. 2c, the relative specific activity of OpSat1p (0.05 mU/mg) was approximately 300-fold lower than that of CysE (17.2 mU/mg). The *in vitro* assay result supported the enzymatic function of OpSat1p as SAT, which converts serine into OAS, although at low level compared to CysE.

As previously reported, the addition of cysteine at various concentrations to the enzyme mixture revealed that the activity of *E. coli* CysE was subjected to feedback inhibition by cysteine (Fig. 2d)^5^. The activity of CysE was dramatically decreased to approximately 40% in the presence of 1 μM cysteine, and the activity further decreased to 10% in the presence of 10 μM cysteine. In contrast, the activity of OpSat1p appeared to be relatively insensitive to the presence of cysteine, implying much less feedback inhibition by cysteine compared to *E. coli* CysE (Fig. 2e). The concentration of cysteine at 50% inhibition of OpSat1p activity was approximately 250 μM, and 1,000 μM cysteine caused the activity to decrease to 30%. Altogether, the data suggest that, compared to the cysteine-sensitive CysE, OpSat1p shows marginal feedback inhibition by cysteine.

### Functional exchange of *E. coli* and *O. parapolymorpha* SATs for sulfur assimilation

**Figure 3:**
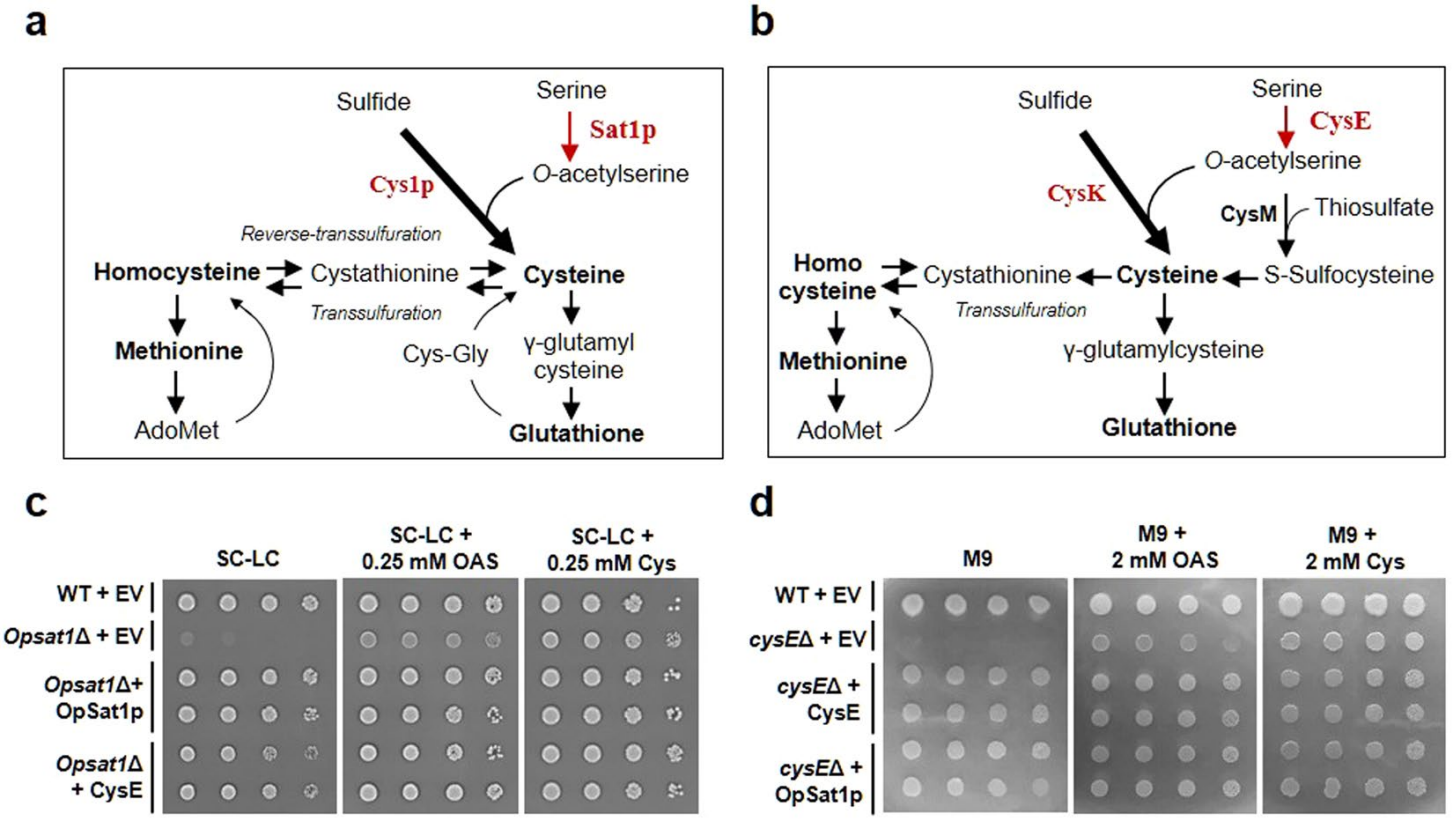
Functional exchange of *Escherichia coli* and *Ogataea parapolymorpha* SATs. (**a**,**b**) Cysteine biosynthesis pathways of *O. parapolymorpha* and *E. coli*, respectively. (**c**,**d**) Complementation analysis of the cysteine auxotrophic phenotypes of *O. parapolymorpha sat1*Δ and *E. coli cysE*Δ. Cells of wild-type *O. parapolymorpha* harboring an empty vector AMIpL1 (WT + EV), *Op*s*at1*Δ harboring an empty vector AMIpL1 (*Op*s*at1*Δ + EV), *Op*s*at1*Δ harboring the OpSat1p expression vector AMIpL1-OpSAT1 (*Op*s*at1*Δ + OpSat1p), and the CysE expression vector AMIpL1-cysE (*Op*s*at1*Δ + CysE) were spotted onto SC-LC plates supplemented with and without 0.25 mM OAS or cysteine. Cells of *E. coli* K-12 BW25113 harboring an empty vector pGEX4T1 (WT + EV), *cysE*Δ harboring an empty vector  pGEX4T1 (*cysE*Δ + EV), *cysE*Δ harboring the CysE expression vector pGEX4T1-cysE (*cysE*Δ + CysE), and *cysE*Δ harboring the OpSat1p expression vector pGEX4T1-OpSAT1 (*cysE*Δ + OpSat1p) were spotted onto M9 minimal medium plates with and without 2 mM OAS or cysteine. All plates were incubated at 37 °C for 2 d.

*O. parapolymorpha* cannot incorporate sulfur into homocysteine due to the absence of *O-*acetylhomoserine sulfhydrylase (encoded by *MET17* in *S. cerevisiae*)^13^. Thus, in *O. parapolymorpha*, cysteine biosynthesis from sulfide and OAS by Cys1p is the only pathway to produce cysteine from inorganic sulfur, indicating that the Sat1p-mediated OAS synthesis is the critical step for sulfur assimilation (Fig. 3a). *E*. *coli* CysE also carries out the essential function in cysteine biosynthesis from sulfide by forming a cysteine synthase complex with CysK^32^. Moreover, *E. coli* possesses an additional assimilation pathway for the biosynthesis of cysteine from thiosulfate by CysM (*S*-sulfocysteine synthase) using *S*-sulfocysteine as a reaction intermediate (Fig. 3b)^33^. Since OpSat1p has a different structural organization with quite low sequence identity (14.7%) and much lower enzymatic activity than *E. coli* CysE does, we carried out heterologous expression of both proteins in *E. coli cysE*Δ and *O. parapolymorpha sat1*Δ strains to investigate whether *E. coli* and *O. parapolymorpha* SATs can be functionally exchanged (Fig. 3c,d). The expression of the OpSat1p or CysE in heterologous hosts was confirmed by western blot analysis (Fig. S3). The expression of *cysE* rescued the cysteine auxotrophic phenotype of the *Opsat1*Δ strain on SC-LC medium (Fig. 3c). Likewise, the expression of *OpSAT1* fully restored the cysteine auxotrophic phenotype of the *E. coli cysE*Δ strain on M9 minimal medium, indicating that OpSat1p can function as an active SAT in *E. coli* (Fig. 3d). These results clearly show that OpSat1p and CysE can be functionally exchangeable as active SATs despite their different domain structures and remote evolutionary distance.

### Sulfur metabolite analysis of *O. parapolymorpha sat1*Δ null mutant and overexpression strains

**Figure 4:**
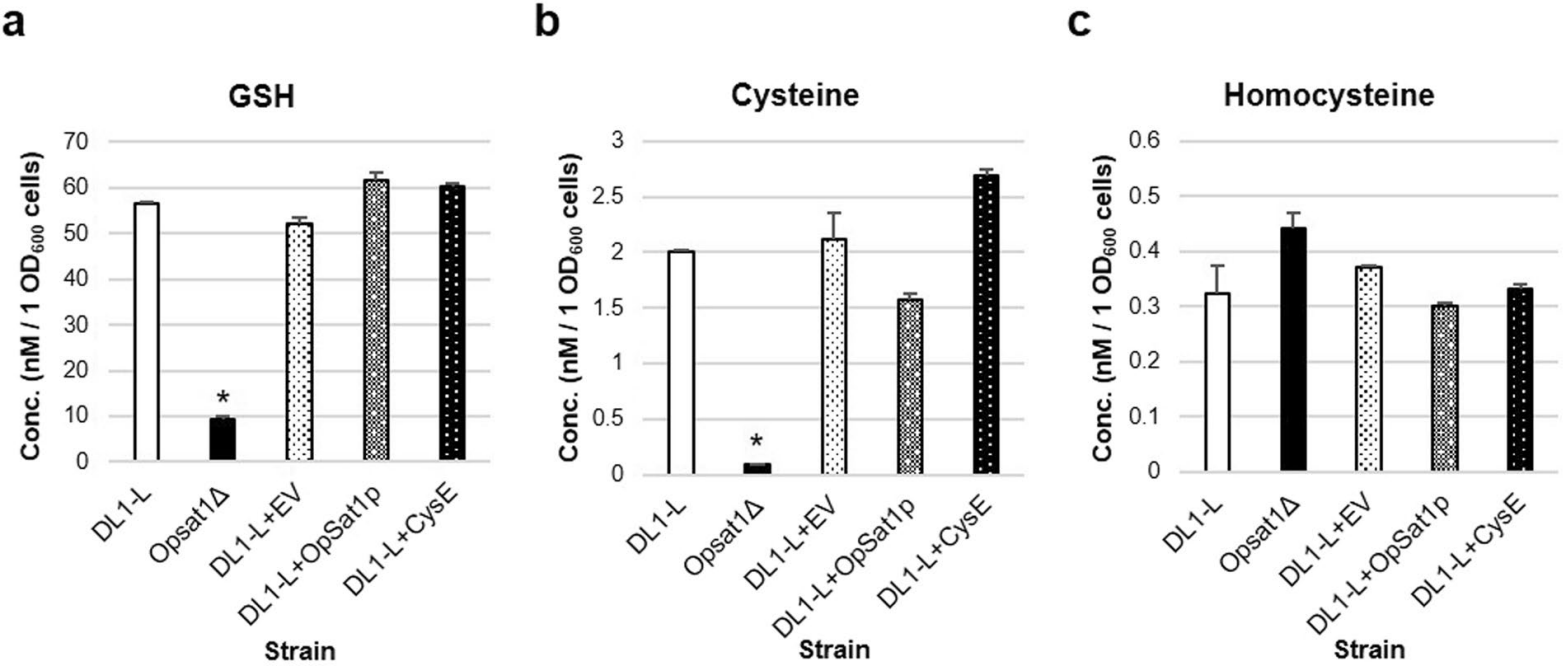
High-performance liquid chromatography (HPLC) analysis of thiol compounds in *Ogataea parapolymorpha* strains. Quantification of (**a**) glutathione (GSH), (**b**) cysteine, and (**c**) homocysteine in *O. parapolymorpha* DL1-L, *Opsat1*Δ, and DL1-L harboring an empty vector (DL1-L + EV), the OpSat1p overexpression vector (DL1-L + OpSat1p), and the CysE overexpression vector (DL1-L + CysE), respectively. Data represent the mean ± SEM of duplicate biological samples. Values were considered to be statistically significant at *p < *0.05 (*). Statistical analysis was taken by the SPSS statistical software (version 25). One-way ANOVA with a Bonferroni post hoc test comparisons was used.

To confirm the effect of *OpSAT1* deletion and overexpression on the intracellular levels of thiol compounds such as GSH, cysteine, and homocysteine in the sulfur assimilatory pathway, HPLC analysis of the metabolites extracted from *O. parapolymorpha* DL1-L, *Opsat1*Δ, DL1-L + EV, DL1-L + *OpSAT1*, and DL1-L + *cysE* strains was performed (Fig. 4). As previously reported, the intracellular level of GSH in wild-type *O. parapolymorpha* was markedly high (56 nM/OD_600_) compared to that of *S. cerevisiae* (2 nM/OD_600_), whereas cysteine and homocysteine showed levels similar to those found in *S. cerevisiae*^34^. In contrast, the deletion of *OpSAT1* directly resulted in cysteine and GSH shortage but not of homocysteine, supporting the hypothesis of OAS synthesis blockage in *Opsat1*Δ. The weak enzymatic activity of OpSat1p (Fig. 2) and the relatively low transcript level of *OpSAT1* (Fig. S4) imply that the *de novo* cysteine biosynthesis via the OAS pathway might be a rate-limiting step in providing sulfur compounds in *O. parapolymorpha*. Thus, we expected that the overexpression of SAT in *O. parapolymorpha* would increase the production of cysteine and GSH. However, the recombinant *O. parapolymorpha* strains overexpressing *OpSAT1* and *cysE* under the same strong *GAP* promoter did not show noticeable differences in the intracellular levels of both cysteine and GSH, although the transcript level of *OpSAT1* under the *GAP* promoter was 10-fold higher than that induced by the native promoter (Fig. S4). This indicated that overexpression of the *SAT* gene alone is not sufficient to increase the intracellular levels of cysteine and GSH in *O. parapolymorpha*.

### Mitochondrial localization of OpSat1p

**Figure 5:**
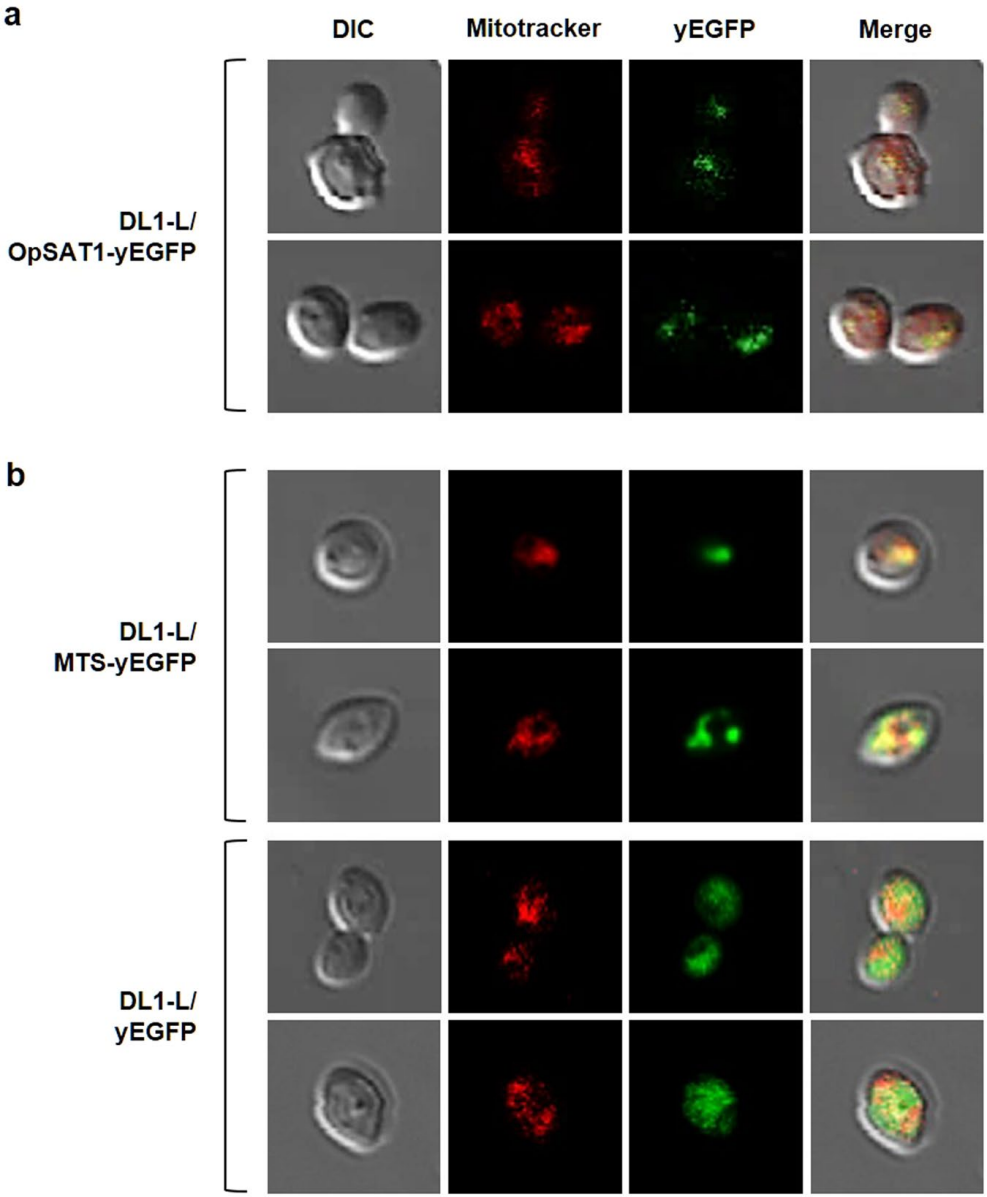
OpSat1p cellular localization analysis. (**a**) Mitochondria-targeted expression of OpSat1p-yEGFP fusion protein. (**b**) Functional analysis of the mitochondrial targeting sequence (MTS) derived from OpSat1p. OpSat1p-yEGFP, MTS-yEGFP, and yEGFP are expressed under the control of the *OpSAT1* native promoter in *O. parapolymorpha* DL1-L. Mitochondria were stained with MitoTracker Red CMXRos (red fluorescence).

The presence of an N-terminal MTS (31 amino acids) in OpSat1p suggests that this enzyme localizes in the mitochondria. To investigate the subcellular localization of OpSat1p, yEGFP was fused at the C-terminus of OpSat1p. The OpSat1p-yEGFP fusion construct was integrated at the locus of the *OpSAT1* promoter on the chromosome of the *O. parapolymorpha* DL1-L strain (DL1-L/OpSAT1-yEGFP). Confocal microscopy analysis of the DL1-L/OpSAT1-yEGFP strain showed that fluorescence signals, which were merged with the signals generated by cells stained with the MitoTracker Red CMXRos dye, mainly arose from the mitochondria (Fig. 5a). To further validate the function of the MTS in mitochondrial targeting, we expressed yEGFP with and without MTS under the control of the *OpSAT1* promoter. Whereas the *O. parapolymorpha* strain expressing the MTS-fused yEGFP construct (DL1-L/MTS-yEGFP) evidently showed strong fluorescent signals mainly at the mitochondria, the *O. parapolymorpha* strain expressing the yEGFP without MTS fusion (DL1-L/yEGFP) showed fluorescent signals that were diffused over the cytoplasm (Fig. 5b, bottom). Altogether, these results strongly support that OpSat1p is located in the mitochondria of *O. parapolymorpha*.

**Figure 6:**
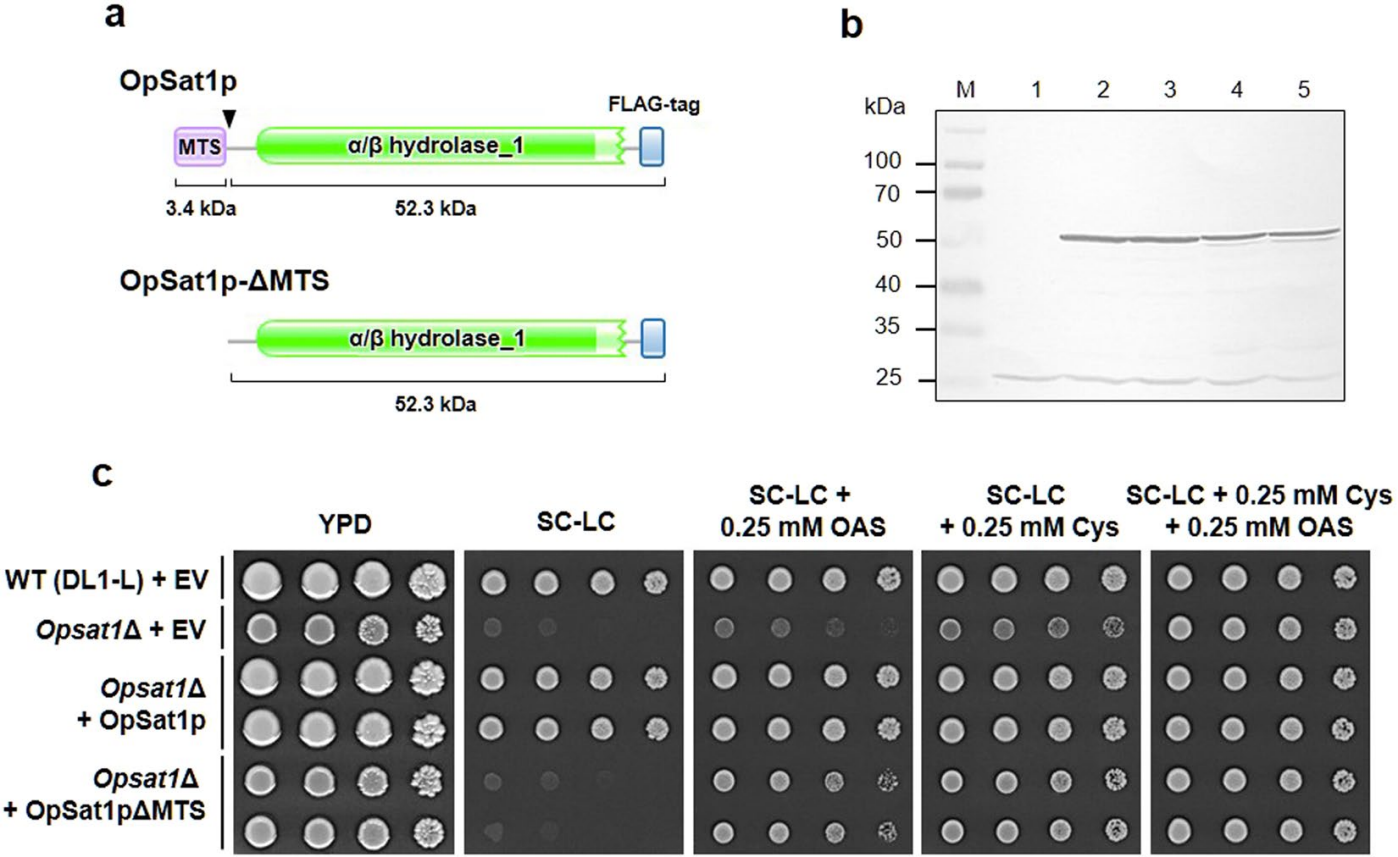
Functional analysis of mitochondrial targeting sequence (MTS)-deleted OpSat1p (OpSat1p-ΔMTS). (**a**) Domain structure of OpSat1p and OpSat1p-ΔMTS. (**b**) Western blot analysis of OpSat1p and OpSat1p-ΔMTS expressed in the *Opsat*1Δ strain. Lane 1, cell lysates of *Opsat1*Δ harboring an empty vector (AMIpL1). Lanes 2 and 3, cell lysates of *Opsat1*Δ harboring the vector AMIpL1-OpSAT1. Lanes 4 and 5, cell lysates of *Opsat1*Δ harboring the vector AMIpL1-OpSAT1(-MTS). (**c**) Growth analysis of the *Opsat*1Δ strain expressing either intact OpSat1p or OpSat1p-ΔMTS. *O. parapolymorpha* cells, including DL1-L + EV, *Opsat1*Δ + EV, *Opsat1*Δ + OpSat1p, and *Opsat1*Δ + OpSat1pΔMTS, were serially diluted and spotted on YPD and SC–LC plates supplemented with 0.25 mM cysteine, OAS, or both. All plates were incubated at 37 °C for 2 d. Yeast cells corresponding to an OD_600_ of 1, 0.1, 0.01 and 0.001 were spotted.

Then, we examined whether the mitochondrial localization of OpSat1p is required for the enzyme activity by expressing the MTS-deleted OpSat1p (OpSat1p-ΔMTS) tagged with FLAG in the *Opsat1*Δ strain (Fig. 6a). Expression of OpSat1p-ΔMTS in the *Opsat1*Δ strain with the expected size of 52.3 kDa was confirmed by western blot analysis using anti-FLAG-mouse antibody (Fig. 6b, lanes 4 and 5). The predicted molecular mass of the precursor OpSat1p retaining MTS is approximately 55.7 kDa, whereas the size of mature OpSat1p (i.e., after MTS cleavage during translocation to the mitochondria) is predicted to around 52.3 kDa. However, the size of mature OpSat1p (Fig. 6b, lanes 2 and 3) was slightly smaller than that of OpSat1p-ΔMTS, indicating that the *in vivo* MTS cleavage might occur a few amino acids away from the predicted 31st amino acid residue. A small, faint band detected just below the major OpSat1p-ΔMTS band (Fig. 6b, lanes 4 and 5) might be a proteolytic form of OpSat1p-ΔMTS, probably generated by altered protein localization due to the lack of MTS. The growth defect of the *OpSAT1* deletion mutant in SC-LC medium was rescued by exogenous supply of either OAS or cysteine, but recovered more efficiently by co-supplementation of both OAS and cysteine. Intriguingly, OpSat1p-ΔMTS could restore OAS auxotrophy, but not cysteine auxotrophy of the *Opsat1*Δ-null mutant strain (Fig. 6c). This indicates that OpSat1p might be functional in the cytoplasm as SAT for the production of OAS, but the mitochondrial localization is required to ensure full activity in cysteine biosynthesis.

## Discussion

Cysteine plays crucial roles not only in the structure, stability, and catalytic function of many proteins, but it is also an important amino acid in the pharmaceutical, food, and cosmetic industries^2,35^. Especially, cysteine is a key precursor of GSH, which has attracted attention as an antioxidant, a detoxifier of xenobiotics, and as an immune booster^36^. Due to its importance and applications, overproduction of cysteine has been attempted in several microorganisms, especially in *E coli*^37,38^. Moreover, since cysteine is involved in survival and virulence of pathogens such as protozoan parasites, it attracted much attention as an alternative drug target^11^, raising the need of understanding its biosynthetic pathway and regulation. In this study, we characterized the structure and function of a novel mitochondrial SAT, essential for *de novo* cysteine biosynthesis in the thermotolerant methylotrophic yeast *O. parapolymorpha*.

Our bioinformatics analysis of SATs from various organisms showed several unique features of yeast and fungal SATs, including OpSat1p. Although bacterial and plant SATs contain an N-terminal SAT domain and hexapeptide repeats at the C-terminus, fungal SATs possess a distinct domain structure consisting of an α/β hydrolase sequence and MTS at the N-terminus (Fig. 1). Noticeably, yeast SATs rather show high similarity to bacterial, yeast, and fungal homoserine *O*-acetyltransferases (HATs), which also contain an α/β hydrolase domain. Based on such markedly different structures compared to their bacterial homologs, it was proposed that fungal SATs might have an independent evolutionary origin by diverging from an ancestor gene encoding a bacterial HAT and, subsequently, acquiring a novel SAT function^20^. The extended phylogenetic tree, including not only fungal and classical SATs but also fungal HATs, supports the proposal that although fungal SATs appeared to be closely related to fungal HATs by sharing the α/β hydrolase domain, they have been early diverged from fungal HATs and evolved separately (Fig. S5).

It is noteworthy that purified recombinant OpSat1p displayed far lower activity than the bacterial homolog CysE did. Furthermore, OpSat1p is much less sensitive to cysteine feedback inhibition than CysE (Fig. 2). In *A*.  *thaliana*, a compartment-specific inhibition by cysteine was observed with cytosolic AtSAT5, whereas other isoforms localized in mitochondria and plastid were insensitive to cysteine^7^. Isoform-specific feedback inhibition of SATs was also demonstrated in the protozoan parasite *E. histolytica* harboring three cytosolic SAT isotypes. Among those, EhSAT3 was fully active even in the presence of cysteine, whereas EhSAT1 and EhSAT2 showed high and moderate sensitivity to cysteine, respectively^39^. However, only SATs in the plastids of pea and spinach are regulated by cysteine^40,41^, suggesting that feedback inhibition of SATs by cysteine may not be tightly associated with the subcellular compartment and may not be a feature in various organisms^42^. Despite such substantial differences in domain structure, enzymatic activity, feedback inhibition, and cellular localization, both bacterial and yeast SATs complemented the cysteine auxotrophy of *Opsat1*Δ- or *cysE*Δ*-*null mutant strains, indicating that OpSat1p is a functional homolog of CysE (Fig. 3). However, considering that OpSat1p lacking MTS failed to restore the growth of the *Opsat1*Δ-null mutant in the absence of cysteine (Fig. 6c), mitochondrial localization of OpSat1p appeared to be vital for its full function in *O. parapolymorpha*. It is noteworthy that whereas the growth of *Opsat1*Δ was not fully restored by addition of OAS, the growth was much improved by the expression of OpSat1p lacking MTS. It is thus speculated that the presence of OpSat1p, regardless of its location, might facilitate the full function of cysteine synthase to convert OAS to cysteine. In contrast, *E. coli* CysE recovered the cysteine auxotrophy of *Opsat1*Δ, implying a different cellular localization requirement between *E. coli* CysE and *O. parapolymorpha* Sat1p for full activity in cysteine biosynthesis.

Intriguingly, all the fungal SATs analyzed for domain structure in this study were predicted to possess an MTS at their N-terminus, which raises the question of what is the significance of SAT mitochondrial localization in fungal species. In plants, mitochondria are the most important compartment for the synthesis of OAS, the precursor of cysteine^8^. As in plants, fungi generate sulfide as an essential intermediate of the sulfur assimilation pathway. Additionally, iron-sulfur cluster turnover might also contribute to the release of sulfide in mitochondria. However, sulfide is a potent inhibitor of cytochrome c oxidase in mitochondria. Thus, efficient sulfide detoxification mechanisms are required to ensure adequate energy production and, consequently, survival of the plant and fungal cells. Therefore, it is proposed that one of the physiological roles of OAS is to catalyze sulfide detoxification in plant mitochondria in addition to cysteine synthesis for sulfur assimilation metabolism^43^. Another possible benefit of mitochondrial localization might be linked to the intracellular acetyl-CoA levels, the substrate of SATs. The concentration of mitochondrial acetyl-CoA is 20- to 30-fold higher than that in the nucleocytosolic compartment in *S. cerevisiae*^44^. Considering the low enzymatic activity of mitochondrial SATs, localization in the mitochondria could be a strategy to transfer acetyl groups to serine from plenty of acetyl-CoA more efficiently than it occurs in the cytoplasm.

It is known that SAT and cysteine synthase (CS), also called *O*-acetyl-L-serine sulfhydrylase (OASS), form an enzyme complex in some plants, such as chives, spinach, and *A. thaliana*^16,45,46^, but apparently not in all of them, as it was reported for *Datura innoxia*^47^. Similarly, SAT and OASS (CS) exist as an enzyme complex in *S. typhimurium* and *E. coli*^5,48,49^ but not in *Brucella abortus* due to structural differences in OASS that interfere with the entry of the SAT C-terminal tail into the active-site pocket^50^. CS-SAT complex formation also varies in parasites. For example, a stable complex between CS and SAT was demonstrated in *L. donovani*, while CS-SAT interaction was not observed in *E. histolytica* due to differences in the C-terminal region, which is involved in complex formation^51^. The complex was not observed in *Trichomonas vaginalis* either, due to the absence of the *SAT* gene^52^. Thus, CS-SAT interaction is not an essential and universal mechanism, and is affected by species-specific differences^11^. Since fungal SATs lack the C-terminal hexapeptide repeat domain required for complex formation, further studies are required to investigate the possibility of CS-SAT interaction in yeast and filamentous fungi.

In terms of industrial applications, overproduction of cysteine and cystine, the oxidized dimer form of cysteine, reached approximately 300 mg/L in wild-type bacterial strains and 990 mg/L in cysteine non-utilizing strains, which were genetically desensitized to feedback inhibition by cysteine^37,53^. In plants, SAT, which has low activity, is the rate-limiting enzyme in the tight regulation of OAS (i.e., CS substrate). Thus, supplementation of OAS to chloroplasts in transgenic plants or overexpression of bacterial or plant SATs prominently enhanced cysteine synthesis. The simultaneous overexpression of both CS and SAT led to total GSH levels higher in most transgenic plants than overexpression of single genes did^54,55^. In our study, overexpression of *OpSAT1* or *cysE* alone did not increase GSH production (Fig. 4). It was also reported that the simultaneous expression of *E. coli cysK*, encoding OASS, along with *cysE* implemented an additional *de novo* cysteine synthetic pathway in *S. cerevisiae*, which subsequently led to increased GSH production^34^.

In conclusion, we present the first report revealing the unique domain organization, enzymatic function, and mitochondrial localization of a novel fungal SAT. Considering its key role in the regulation of the cysteine biosynthesis pathway in *O. parapolymorpha*, OpSat1p could be a rational target of metabolic engineering to generate yeast cell factories producing high-valued, sulfur-containing metabolites, such as GSH.

## Materials and Methods

### Strains and culture media

The *O. parapolymorpha* and *E. coli* strains used in this study are listed in the Supplementary Table S1. *O. parapolymorpha* cells were grown at 37 °C in YPD (1% yeast extract, 2% bacto-peptone and 2% dextrose) or SC-LC medium (0.67% yeast nitrogen base without amino acids, 2% glucose, amino acids and base mixture lacking leucine and cysteine). *E. coli* cells were grown in LB broth (0.5% bacto-yeast extract, 1% bacto-tryptone, 1% NaCl) with or without 100 μg/mL ampicillin. For analysis of the cysteine auxotrophic phenotype, M9 minimal medium (12 g of Na_2_HPO_4_, 3 g of KH_2_PO_4_, 0.5 g of NaCl, 1 g of NH_4_Cl per liter, 2 mM MgSO_4_, 0.1 mM CaCl_2_, 0.4% glucose, 0.02 mM IPTG (isopropyl β-D-thiogalactopyranoside), 1 mg/mL thiamine) was used.

### Construction of SAT expression vectors

The plasmid and primer sets used in this study are listed in the Supplementary Table S2. To construct bacterial vectors expressing SAT as a glutathione S-transferase (GST)-fusion protein, the *OpSAT1* open reading frame (ORF) was amplified by polymerase chain reaction (PCR) using the primer set EcoRI OpSAT1 1F (w/o ATG)/OpSAT1 6His 2B Stop SalI and the genomic DNA of the *O. parapolymorpha* DL1-L strain. The *cysE* ORF was amplified by PCR using the primer set EcoRI cysE 1F (w/o ATG)/cysE 6His 2B Stop SalI and pCysE as the template. Each PCR product was cloned between the *Eco*RI and *Sal*I sites of the pGEX4T1 plasmid, generating pGEX4T1-OpSAT1 and pGEX4T1-cysE. To construct an *O. parapolymorpha* vector expressing a FLAG-tagged OpSat1p, the *OpSAT1* ORF region was amplified by PCR using the primer set EcoRI-OpSAT1-1F/OpSAT1-2B-FLAG18. The resulting PCR product was used as the template for the second PCR round, using the primer set EcoRI-OpSAT1-1F/FLAG-Stop-SalI to generate the OpSAT1-FLAG fragment, which was ligated with the *Eco*RI/*Sal*I-digested AMIpL1 plasmid, resulting in the *O. parapolymorpha* vector AMIpL1-OpSAT1. The *O. parapolymorpha* vector AMIpL1-cysE expressing His-tagged CysE was constructed by sub-cloning the *cysE* DNA fragment, which was amplified by the primer set EcoRI cysE 1F/cysE 6His 2B Stop SalI, into the *Eco*RI/*Sal*I-digested AMIpL1.

### Purification and SAT activity assay of recombinant GST-fusion SATs

The recombinant *E. coli* BL21 (DE3) cells harboring pGEX4T1-OpSAT1 or pGEX4T1-cysE were cultivated until optical dispersion at 600 nm (OD_600_) = 0.5. IPTG (0.02 mM) was added to induce SAT expression and cells were additionally cultured for 4 h at 37 °C with shaking. *E. coli* cells were collected and resuspended with lysis buffer containing 20 mM sodium phosphate, pH 7.4, 0.5 M NaCl, 40 mM Imidazole, 1× PIC (protease inhibitor cocktail, SIGMA), and 1 mM PMSF (phenylmethyl sulfonyl fluoride). The cells were broken by sonication on ice (30 s × 20 repeats) and the lysates were collected by centrifugation at 16,000 rpm for 15 min at 4 °C. The supernatant was filtered with a 0.45 μm filter and mixed with 2 mL of Ni-Sepharose 6 Fast Flow resin (GE Healthcare) in a column. The mixture was incubated at 4 °C for at least 30 min and washed thrice with 4 mL of binding buffer (20 mM sodium phosphate, pH 7.4, 0.5 M NaCl, and 40 mM imidazole). His-tagged proteins bound to the Ni^2+^ column were eluted with elution buffer (20 mM sodium phosphate, pH 7.4, 0.5 M NaCl, and 500 mM imidazole). The purified proteins were subsequently concentrated using an Amicon Ultra Centrifugal Filters Ultracel-30K (Millipore). Concentrated proteins were quantified using a NanoDrop Microvolume Spectrophotometer (ACTGene). Activity of SATs was analyzed based on the decrease of OD_232_ caused by the hydrolysis of the thioester bond in acetyl-CoA, as previously described with some modifications^49^. Briefly, the enzyme mixture for the SAT assay contains 100 mM potassium phosphate buffer, pH 7.4, 1 mM L-serine, 0.1 mM acetyl-CoA, and purified CysE or HpSat1p in a final volume of 400 μL in cuvettes of 10 mm path length at room temperature. For feedback inhibition assay of SAT activity, L-cysteine at various concentrations was added in the enzyme mixture. Enzyme activity was assayed in triplicate.

### Microscopy analysis of green fluorescence protein (GFP)-fusion proteins

The yeast enhanced GFP (yEGFP) construct, yEGFP construct fused with the mitochondrial targeting sequence (MTS-yEGFP), yEGFP fused with the whole *OpSAT1* sequence (OpSAT1-yEGFP), which were all expressed under the control of the *OpSAT1* native promoter, were constructed as follows. The DNA fragments of the *yEGFP* ORF (BglII yEGFP 1F/ter yEGFP 2B KpnI), native promoter of *OpSAT1* (SphI pOpSAT1 1F/pOpSAT1 2B BglII), native promoter of *OpSAT1* with MTS sequence (SphI pOpSAT1 1F/OpSAT1MTS 3B Gly5 BglII), and *OpSAT1* with its native promoter (SphI pOpSAT1 1F/OpSAT1 2B BglII) were generated by PCR using each primer set. The three PCR-amplified fragments (the *OpSAT1* native promoter, the *OpSAT1* promoter with MTS sequence, and the *OpSAT1* ORF with its native promoter) were ligated with the *yEGFP* ORF fragment and cloned between the *Sph*I and *Kpn*I sites of the pT-HpLEU2-NS(c) plasmid, generating pT-SAT1(p)-yEGFP, pT-SAT1(p)-MTS-yEGFP, and pT-OpSAT1-yEGFP, respectively. The GFP-fusion plasmids were linearized by digestion with *Hin*dIII and integrated into the chromosomal *OpSAT1* locus. The resulting strains were grown overnight at 37 °C in 2 mL of YPD. Pre-cultured cells were inoculated with initial OD_600_ = 0.3 in 5 mL of YPD medium and incubated for 4 h. After cultivation, MitoTracker Red CMXRos stain (Molecular probes, Eugene) was added to a final concentration of 15 nM in the YPD medium for mitochondria staining, and the cells were incubated in a rotator at 25 rpm for 30 min. Cells were washed 3 times in 1× phosphate-buffered saline (PBS) buffer and harvested. Cell pellet was resuspended in 1.2 mL of PBS containing 3.7% formaldehyde and incubated in a rotator at 25 rpm for 10 min to fixation. After fixation, cells were washed 3 times in 1 mL of 1× PBS and resuspended in 1× PBS. Imaging of the prepared cell was performed using a confocal microscope (Zeiss LSM 700).

### High-performance liquid chromatography (HPLC) analysis of thiol compounds

Yeast metabolites were extracted from 10 mL of each culture (20 A_600_ units of cells). Briefly, washed yeast cells were plunged into 1 mL of methanol to inactivate enzymes. Methionine sulfone was added to a concentration of 5.6 μM as an internal cationic standard. The solution was incubated for 5 min at room temperature. Then, 1 mL of chloroform and 380 μL of Milli-Q water were added to the solution, and the mixture was thoroughly mixed to remove phospholipids liberated from cell membranes. The separated 1 mL methanol layer was centrifugally filtered through a Millipore 3 kDa-cutoff filter to remove proteins. The filtrate was lyophilized and dissolved in 60 μL of Milli-Q water before HPLC analysis. The thiol compounds in the extracted metabolites were labeled with 4-fluoro-7-sulfobenzofurazan and analyzed using a Cosmosil 5C18-PAQ column (4.6 × 250 mm, Nacalai Tesque) under HPLC analysis conditions previously described^31^. Standard curves based on the peak area of GSH (SIGMA), homocysteine (SIGMA), and cysteine (SIGMA) were used for quantification of thiol compounds.

## Electronic supplementary material

Supplementary information
