# Supplementary material for: A Novel Mitochondrial Serine O-Acetyltransferase, OpSAT1, Plays a Critical Role in Sulfur Metabolism in the Thermotolerant Methylotrophic Yeast Ogataea parapolymorpha
Source: Sci Rep. 2018 Feb 5;8:2377. doi: 10.1038/s41598-018-20630-8 (PMC5799214; doi:10.1038/s41598-018-20630-8)
Supplement: Supplementary file 1 — Supplementary information [file 41598_2018_20630_MOESM1_ESM.docx]

**Supplementary Information**

**A Novel Mitochondrial Serine *O*-Acetyltransferase, OpSAT1, Plays a Critical Role in Sulfur Metabolism in the Thermotolerant Methylotrophic Yeast *Ogataea parapolymorpha***

Ji Yoon Yeon^1#^, Su Jin Yoo^1#^, Hiroshi Takagi^2^* and Hyun Ah Kang^1^*

^1^ Department of Life Science, Chung-Ang University, Seoul 06974, Korea, ^2^ Graduate School of Biological Sciences, Nara Institute of Science and Technology, Nara 630-0192, Japan

#These authors contributed equally

*Correspondence e-mail: [hiro@bs.naist.jp](mailto:hiro@bs.naist.jp), [hyunkang@cau.ac.kr](mailto:hyunkang@cau.ac.kr)

**Contents**

**Supplementary Figures S1-S5**

**Supplementary Tables S1-S3**

**Supplementary Figure 1**

**
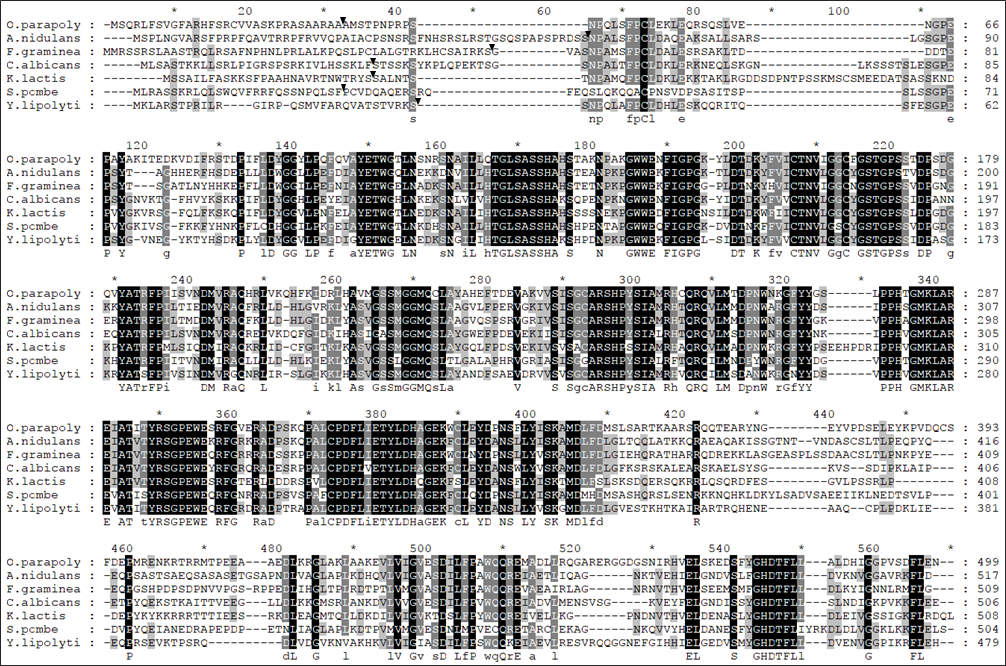
**

**Fig. S1.** Protein sequence alignment of OpSat1p with other fungal SATs. Accession numbers are: *Ogataea parapolymorpha* (ESW96161), *Aspergillus nidulans* (LT613643), *Fusarium graminearum* (ESU05317), *Candida albicans* (XP_713026), *Kluyveromyces lactis* (XP_456042), *Schizosaccharomyces pombe* (CAB53733), and *Yarrowia lipolytica* (AOW03346). The alignment was obtained using the ClustalW software. Conserved regions are shaded in black and similar regions are shaded in grey. Putative cleavage sites for mitochondrial pre-sequences are marked by arrowheads (▼).

**Supplementary Figure 2**

**
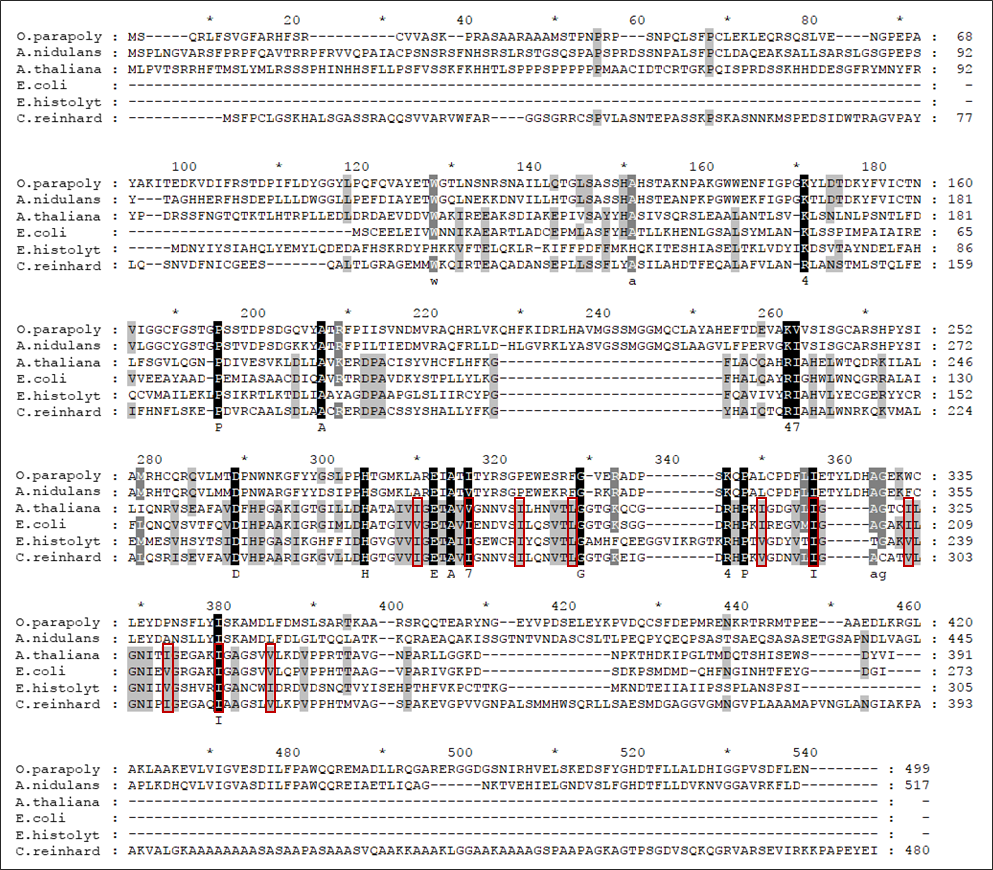
**

**Fig. S2.** Protein sequence alignment of representative SATs from various organisms. Accession numbers are: *O. parapolymorpha* (ESW96161), *A. nidulans* (LT613643), *Arabidopsis thaliana* SAT-m (U22964), *Escherichia coli* (AAC76631), *Entamoeba histolytica* (AB023954), *Chlamydomonas reinhardtii* (EDO98094). Conserved regions are shaded in black and similar regions are shaded in grey. The first amino acids Val, Leu, or Ile, followed often by Gly in the hexapeptide repeats with a consensus sequence of {V,L,I}-G-X-X-X-X^1,2^, are marked with red boxes in the classical SAT proteins.

**Supplementary Figure 3**

**
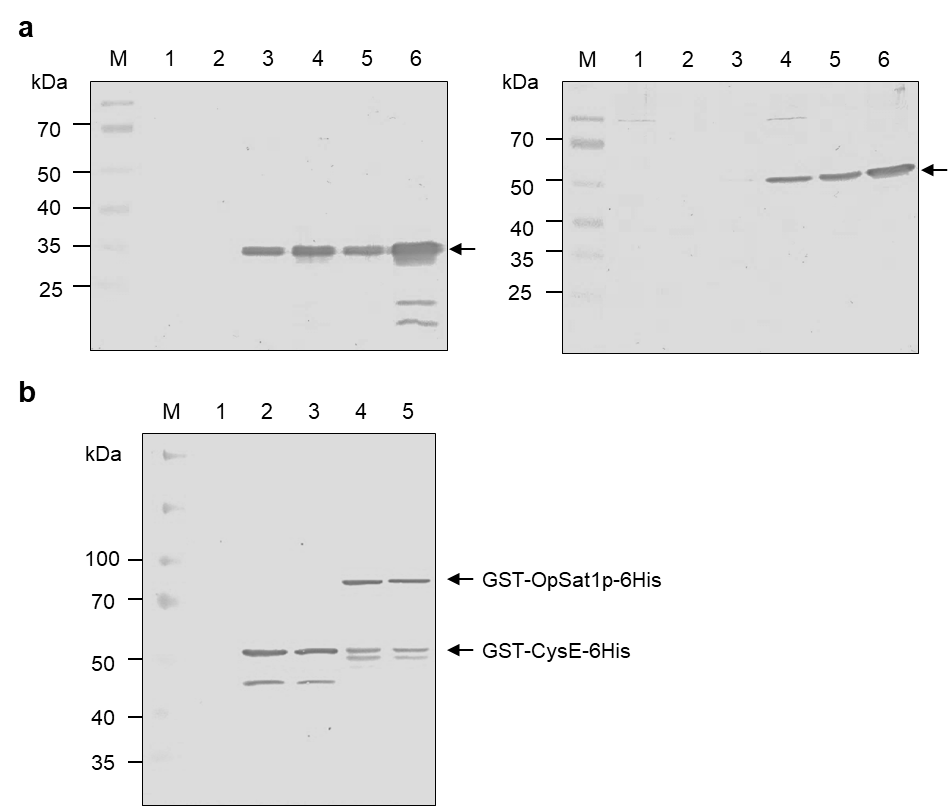
**

**Fig. S3.** Western blot analysis of CysE and OpSat1p expressed in the *O. parapolymorpha* DL1-L and *sat1*Δ strains, respectively, for complementation test. **(A)** Analysis of CysE and OpSat1p expressed in the *O. parapolymorpha* DL1-L and *sat1*Δ strains. Lanes 1 and 2, cell lysates of wild-type *O. parapolymorpha* and *sat1*Δ harboring an empty vector (AMIpL1). Lanes 3 and 4, cell lysates of DL1-L cells harboring AMIpL1-cysE or AMIpL1-OpSAT1. Lanes 5 and 6, cell lysates of *Opsat1*Δ harboring AMIpL1-cysE or AMIpL1-OpSAT1. Anti-His-Mouse or Anti-FLAG-mouse antibody (1:2000) was used as primary antibody. Anti-mouse-AP (1: 10000) was used as secondary antibody. Sizes of the CysE-6His and OpSat1p-FLAG are approximately 30.7 kDa and 52.3 kDa, respectively. **(B)** Western blot analysis of GST-CysE and GST-OpSat1p expressed in the *E. coli cysE*Δ strain. Lane 1, cell lysates of *E. coli cysE*Δ harboring an empty vector (pGEX4T1). Lanes 2 and 3, cell lysates of *E. coli cysE*Δ harboring pGEX4T1-cysE. Lanes 4 and 5, cell lysates of *E. coli cysE*Δ harboring pGEX4T1-OpSAT1. Anti-His-mouse antibody (1: 2000) was used as primary antibody and anti-mouse-AP antibody (1: 10000) was used as secondary antibody. Sizes of the GST-CysE and GST-OpSat1p are approximately 55.9 kDa and 80.6 kDa, respectively.

**Supplementary Figure 4**


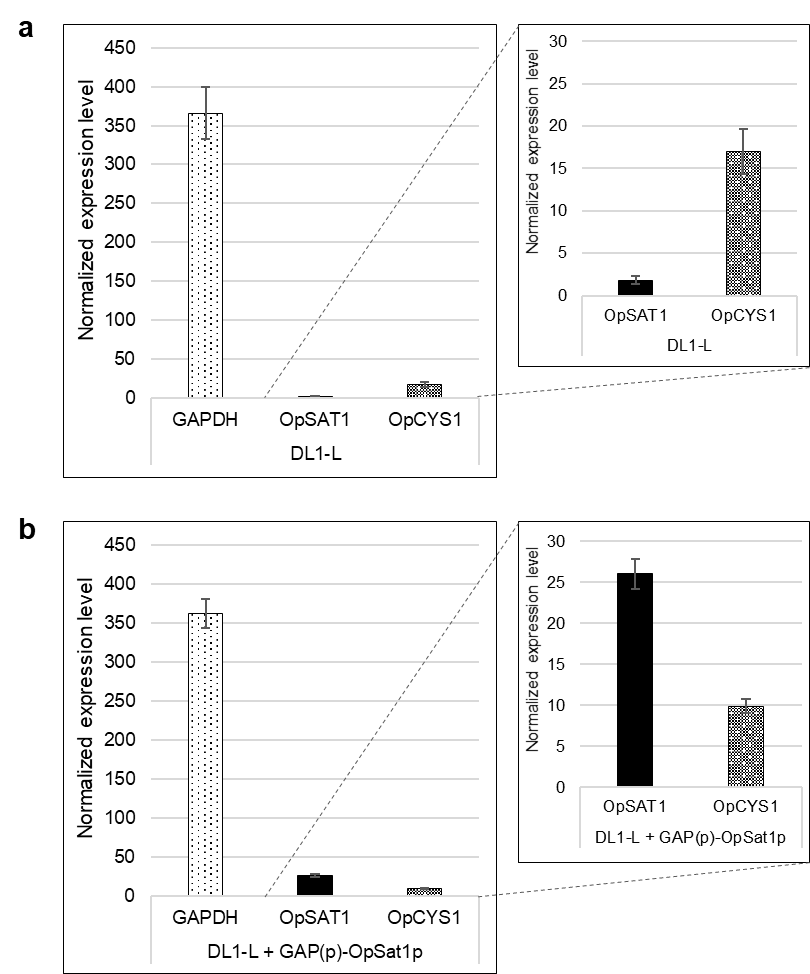


**Fig. S4.** Quantitative reverse transcription polymerase chain reaction (qRT-PCR) analysis of the transcript levels of *OpSAT1* and *OpCYS1* in *O. parapolymorpha*. Total RNA was isolated from the *O. parapolymorpha* DL1-L strain and DL1-L strain harboring the *OpSAT1* overexpression plasmid (AMIpL1-OpSAT1) and *cysE* overexpression plasmid (AMIpL1-cysE), respectively. Yeast cells at OD_600_ = 0.1 were grown to 0.5 in YPD at 37°C for total RNA preparation. cDNA was synthesized using an RnaUsScript reverse transcriptase kit (LeGene Biosciences). qRT-PCR was performed in duplicate using SYBR Premix Ex Taq (Tli RNaseH Plus, Takara) and CFX96 Real-Time PCR detection system (Bio-Rad). Normalized fold expression [∆∆C(q)] was calculated with the CFX manager software using *ACT1* as the reference gene. All primers used in qRT-PCR analysis are listed in Table S3.

**Supplementary Figure 5**

**
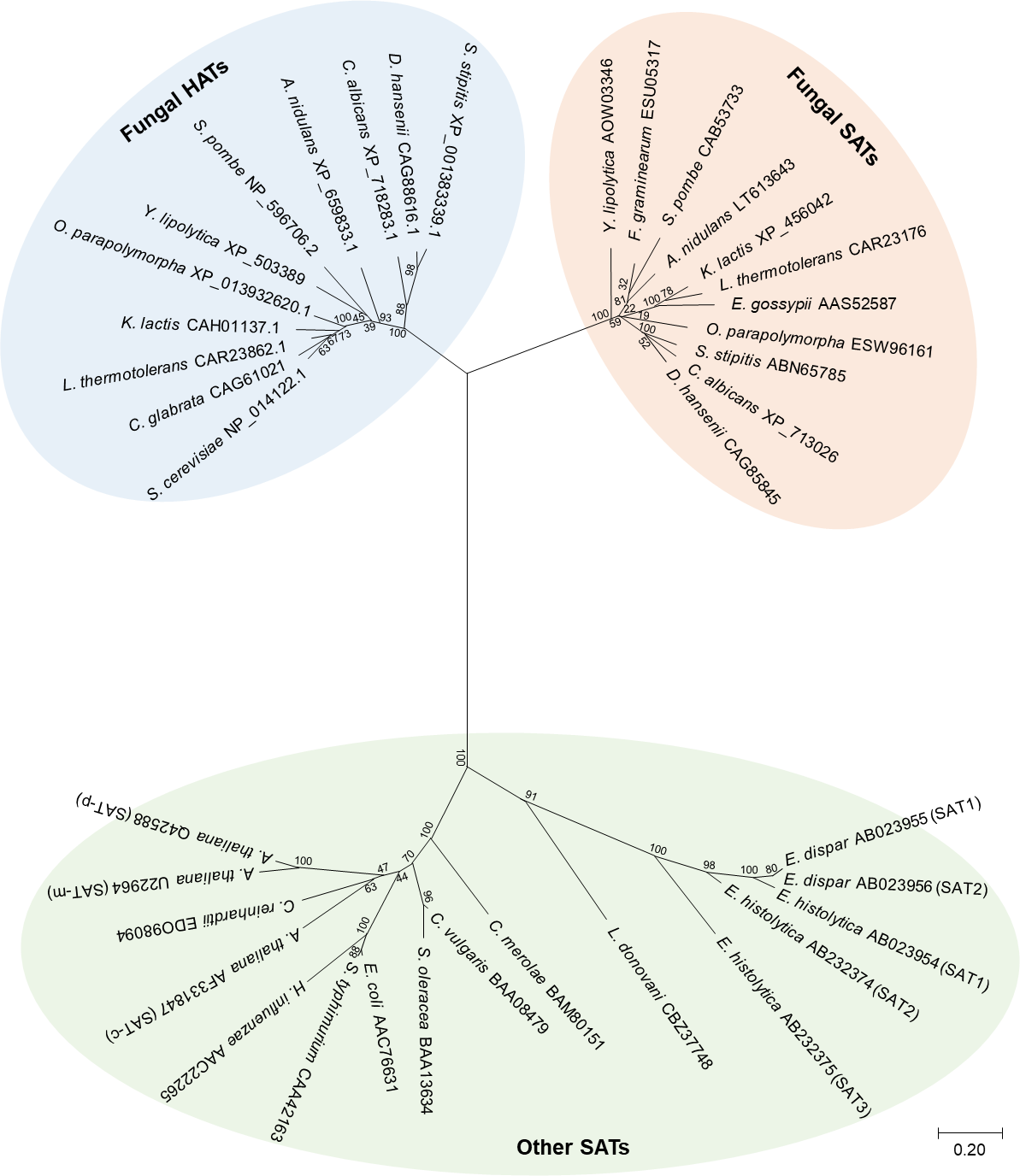
**

**Fig. S5.** Phylogenetic tree of SATs from various organisms and fungal HATs (homoserine *O*-acetyl transferase). The phylogenetic tree was constructed including twenty-seven SAT proteins from representative organisms and eleven fungal HAT proteins. Amino acid sequences of SAT were obtained from the GenBank database and subjected to phylogenetic tree construction using the Neighbor joining (NJ) method built in the MEGA7 software. GenBank accession numbers are indicated and some isoforms are written in parenthesis. The scale bar represents 0.2 amino acid substitutions per site. Bootstrap values (100 replicates) are shown next to the branches.

**Supplementary Table S1.** Strains used in this study

| **Strain** | **Genotype** | **Source or reference** |
| --- | --- | --- |
| ***Escherichia coli*** | | |
| BW25113 | *lacI*^q^ *rrnB*_T14_ Δ*lacZ*_WJ16_ *hsdR514* Δ*araBAD*_AH33_ Δ*rhaBAD*_LD78_ | Datsenko *et al*.^3^ |
| BW25113 + pCysE | BW25113 pCysE::Cm^R^ | Kitagawa *et al.*^4^ |
| BW25113 + EV | BW25113 harboring pGEX4T1 | This study |
| JW3582 | BW25113 Δ*cysE* | Baba *et al.*^5^ |
| JW3582 + EV | BW25113 Δ*cysE* harboring pGEX4T1 | This study |
| JW3582 + OpSat1p | BW25113 Δ*cysE* harboring pGEX4T1-OpSAT1 | This study |
| JW3582 + CysE | BW25113 Δ*cysE* harboring pGEX4T1-cysE | This study |
| BL21(DE3) | F^–^*dcm ompT hsdSB* (rB^–^mB^–^) *gal* | Invitrogen |
| BL21(DE3) + OpSat1p | BL21(DE3) harboring pGEX4T1-OpSAT1 vector | This study |
| BL21(DE3) + CysE | BL21(DE3) harboring pGEX4T1-cysE vector | This study |
| ***Ogataea parapolymorpha*** | | |
| DL1-L | *leu2* | Kang *et al.*^6^ |
| DL1-L/OpSAT1-yEGFP | DL1-L harboring pT-OpSAT1-yEGFP vector | This study |
| DL1-L/MTS-yEGFP | DL1-L harboring pT-SAT1(p)-MTS-yEGFP vector | This study |
| DL1-L/yEGFP | DL1-L harboring pT-SAT1(p)-yEGFP vector | This study |
| DL1-L + EV | DL1-L harboring AMIpL1 empty vector | This study |
| DL1-L + OpSat1p | DL1-L harboring AMIpL1-OpSAT1 vector | This study |
| DL1-L + CysE | DL1-L harboring AMIpL1-cysE vector | This study |
| DL1-L + OpSat1pΔMTS | DL1-L harboring AMIpL1-OpSAT1(-MTS) | This study |
| *Opsat1*Δ | *leu2 ura3::lacZ sat1::URA3* | Sohn *et al.*^7^ |
| *Opsat1*Δ + EV | *Opsat1*Δ harboring AMIpL1 vector | This study |
| *Opsat1*Δ + OpSat1p | *Opsat1*Δ harboring AMIpL1-OpSAT1 vector | This study |
| *Opsat1*Δ + CysE | *Opsat1*Δ harboring AMIpL1-cysE vector | This study |

**Supplementary Table S2.** Plasmids used in this study

| **Plasmid** | **Description** | **Reference** |
| --- | --- | --- |
| pGEX4T1 | *Escherichia coli* protein expression vector, Amp^R^, GST-tag | Amersham Pharmacia Biotech |
| pGEX4T1-OpSAT1 | pGEX4T1 vector expressing GST-OpSat1p fusion protein tagged with 6His at C-terminus | This study |
| pGEX4T1-cysE | pGEX4T1 vector expressing GST-cysE fusion protein tagged with 6His at C-terminus | This study |
| AMIpL1 | *Ogataea parapolymorpha* ARS-vector, Amp^R^, *LEU2* | Agaphonov *et al.*^8^ |
| AMIpL1-OpSAT1 | AMIpL1 expressing OpSat1p tagged with a C-terminal FLAG | This study |
| AMIpL1-OpSAT1(-MTS) | AMIpL1 expressing MTS-deleted OpSat1p tagged with a C-terminal FLAG | This study |
| AMIpL1-cysE | AMIpL1 expressing CysE tagged with a C-terminal 6His | This study |
| pT-HpLEU2-NS(c) | *O. parapolymorpha* integration vector, Amp^R^, *LEU2* | Kim *et al.*^9^ |
| pDLMOX-yEGFP | *O. parapolymorpha* ARS-vector expressing yEGFP under the control of the *MOX* promoter | Sohn *et al.*^10^ |
| pT-OpSAT1-yEGFP | pT-HpLEU2-NS(c) expressing OpSAT1-GFP under the control of the *OpSAT1* native promoter | This study |
| pT-SAT1(p)-MTS-yEGFP | pT-HpLEU2-NS(c) expressing GFP fused to MTS derived from OpSat1p under the control of the *OpSAT1* native promoter | This study |
| pT-SAT1(p)-yEGFP | pT-HpLEU2-NS(c) expressing GFP under the control of the *OpSAT1* native promoter | This study |

**Supplementary Table S3.** Primers used in this study

| **Primer** | **Sequence (5'->3')** | **Purpose** |
| --- | --- | --- |
| EcoRI OpSAT1 1F (w/o ATG) | gcgcGAATTCagccagcgtctgttctct | Amplification of *OpSAT1* without ATG |
| OpSAT1 6His 2B Stop SalI | gcgcGTCGACttagtggtggtggtggtggtgattttctaa  aaaatcgctgactg | Amplification of *OpSAT1* for His tagging |
| EcoRI cysE 1F (w/o ATG) | gcgcGAATTCtcgtgtgaagaactggaaa | Amplification of *cysE* without ATG |
| cysE 6His 2B Stop SalI | gcgcGTCGACttagtggtggtggtggtggtggatcccatc  cccatactc | Amplification of *cysE* for His tagging |
| EcoRI cysE 1F | gcgcGAATTCatgtcgtgtgaagaactgga | Amplification of *cysE* |
| EcoRI OpSAT1 1F | cgGAATTCatgagccagcgtctgttc | Amplification of *OpSAT1* |
| OpSAT1 2B FLAG18 | gtcgtcatccttgtaatcattttctaaaaaatcgctgactg | Amplification of *OpSAT1* for FLAG tagging |
| FLAG Stop SalI | gcgcgtcgacttacttatcgtcgtcatccttgtaatc | Amplification of *OpSAT1* for FLAG tagging |
| SphI pOpSAT1 1F | gcgcgcGCATGCctaatatttctccgagtctatg | Amplification of *OpSAT1* for yEGFP fusion |
| OpSAT1 2B BglII | gcgcAGATCTattttctaaaaaatcgctgac | Amplification of *OpSAT1* for yEGFP fusion |
| OpSAT1MTS 3B Gly5 BglII | gcgcAGATCTACCGCCACCGCCACCtga  catagcggccgca | Amplification of MTS from *OpSAT1* |
| pOpSAT1 2B BglII | gcgcAGATCTgggatgggaagaataattgaaag | Amplification of *OpSAT1* promoter |
| BglII yEGFP 1F | gcgcAGATCTatgtctaaaggtgaagaattattc | Amplification of yEGFP |
| ter yEGFP 2B KpnI | gcgcGGTACCatagatgtaatcaacgaggcc | Amplification of yEGFP |
| EcoRI MTS 1F (with ATG) | gcgcGAATTCATGgctatgtcaacccc | Amplification of *OpSAT1* lacking MTS |
| FLAG 2B SalI | gcgcGTCGACcttatcgtcgtcatccttg | Amplification of *OpSAT1* lacking MTS |
| OpACT1 qRT-PCR 1F | tccaggctgtgctgtcgttg | qRT-PCR analysis of *ACT1* |
| OpACT1 qRT-PCR 2B | ccggccaagtcgattctcaa | qRT-PCR analysis of *ACT1* |
| OpGAPDH qRT-PCR 1F | tggatacaccgacgaggctg | qRT-PCR analysis of *GAP* |
| OpGAPDH qRT-PCR 2B | cgagcttgacgaaggttggg | qRT-PCR analysis of *GAP* |
| OpSAT1 qRT-PCR 1F | gtgtacgccacgcggtttc | qRT-PCR analysis of *SAT1* |
| OpSAT1 qRT-PCR 2B | ctcgtgagcgtatgcgagac | qRT-PCR analysis of *SAT1* |
| OpCYS1 qRT-PCR 1F | gagggcattggtcaaggcc | qRT-PCR analysis of *CYS1* |
| OpCYS1 qRT-PCR 2B | gcgctgaagtgcctccaac | qRT-PCR analysis of *CYS1* |

**References**

1 Vaara, M. Eight bacterial proteins, including UDP-*N*-acetylglucosamine acyltransferase (LpxA) and three other transferases of *Escherichia coli*, consist of a six-residue periodicity theme. *FEMS Microbiol Lett* **76**, 249-254 (1992).

2 Vuorio, R., Harkonen, T., Tolvanen, M. & Vaara, M. The novel hexapeptide motif found in the acyltransferases LpxA and LpxD of lipid A biosynthesis is conserved in various bacteria. *FEBS Lett* **337**, 289-292 (1994).

3 Datsenko, K. A. & Wanner, B. L. One-step inactivation of chromosomal genes in *Escherichia coli* K-12 using PCR products. *Proc Natl Acad Sci U S A* **97**, 6640-6645, doi:10.1073/pnas.120163297 (2000).

4 Kitagawa, M. *et al.* Complete set of ORF clones of *Escherichia coli* ASKA library (a complete set of *E. coli* K-12 ORF archive): unique resources for biological research. *DNA Res* **12**, 291-299, doi:10.1093/dnares/dsi012 (2005).

5 Baba, T. *et al.* Construction of *Escherichia coli* K-12 in-frame, single-gene knockout mutants: the Keio collection. *Mol Syst Biol* **2**, 2006 0008, doi:10.1038/msb4100050 (2006).

6 Kang, H. A. *et al.* Development of expression systems for the production of recombinant human serum albumin using the *MOX* promoter in *Hansenula polymorpha* DL-1. *Biotechnol Bioeng* **76**, 175-185 (2001).

7 Sohn, M. J. *et al.* Novel cysteine-centered sulfur metabolic pathway in the thermotolerant methylotrophic yeast *Hansenula polymorpha*. *PLoS One* **9**, e100725, doi:10.1371/journal.pone.0100725 (2014).

8 Agaphonov, M. O. *et al.* Vectors for rapid selection of integrants with different plasmid copy numbers in the yeast *Hansenula polymorpha* DL1. *Yeast* **15**, 541-551, doi:10.1002/(sici)1097-0061(199905)15:7<541::aid-yea392>3.0.co;2-g (1999).

9 Kim, H. A. *et al.* Functional and molecular characterization of novel *Hansenula polymorpha* genes, *HpPMT5* and *HpPMT6*, encoding protein *O*-mannosyltransferases. *Fungal Genet Biol* **58-59**, 10-24, doi:10.1016/j.fgb.2013.08.003 (2013).

10 Sohn, M. J. *et al.* *HpYPS1* and *HpYPS7* encode functional aspartyl proteases localized at the cell surface in the thermotolerant methylotrophic yeast *Hansenula polymorpha*. *Yeast* **29**, 1-16, doi:10.1002/yea.1912 (2012).
